# DACT2 silencing by promoter CpG methylation disrupts its regulation of epithelial-to-mesenchymal transition and cytoskeleton reorganization in breast cancer cells

**DOI:** 10.18632/oncotarget.12341

**Published:** 2016-09-29

**Authors:** Tingxiu Xiang, Yichao Fan, Chunhong Li, Lili Li, Ying Ying, Junhao Mu, Weiyan Peng, Yixiao Feng, Michael Oberst, Kathleen Kelly, Guosheng Ren, Qian Tao

**Affiliations:** ^1^ Chongqing Key Laboratory of Molecular Oncology and Epigenetics, The First Affiliated Hospital of Chongqing Medical University, Chongqing, China; ^2^ Cancer Epigenetics Laboratory, Department of Clinical Oncology, Sir YK Pao Center for Cancer and Li Ka Shing Institute of Health Sciences, The Chinese University of Hong Kong and CUHK Shenzhen Research Institute, Hong Kong; ^3^ Oncology Department, Suining Sichuan Center Hospital, Sichuan, China; ^4^ Signal Transduction Section, National Cancer Institute, National Institutes of Health, Bethesda, Maryland, USA

**Keywords:** DACT2, tumor suppressor, methylation, EMT, breast cancer

## Abstract

Wnt signaling plays an important role in breast carcinogenesis. DAPPER2 (DACT2) functions as an inhibitor of canonical Wnt signaling and plays distinct roles in different cell contexts, with its role in breast tumorigenesis unclear. We investigated DACT2 expression in breast cancer cell lines and primary tumors, as well as its functions and molecular mechanisms. Results showed that DACT2 expression was silenced in 9/9 of cell lines. Promoter CpG methylation of *DACT2* was detected in 89% (8/9) of cell lines, as well as in 73% (107/147) of primary tumors, but only in 20% (1/5) of surgical margin tissues and in none of normal breast tissues. Demethylation of BT549 and T47D cell lines with 5-aza-2'-deoxycytidine restored *DACT2* expression along with promoter demethylation, suggesting that its downregulation in breast cancer is dependent on promoter methylation. Furthermore, ectopic expression of *DACT2* induced breast cell apoptosis *in vitro*, and further inhibited breast tumor cell proliferation, migration and EMT, through antagonizing Wnt/β-catenin and Akt/GSK-3 signaling. Thus, these results demonstrate that DACT2 functions as a tumor suppressor for breast cancer but was frequently disrupted epigenetically in this cancer.

## INTRODUCTION

Breast cancer is a highly heterogeneous cancer associated with alterations in multiple signal pathways. Wnt signaling plays a key role during breast carcinogenesis and progression [1–2]. Wnt signaling pathway is involved in cell proliferation, apoptosis, differentiation, motility, and survival in a context-dependent manner [3].

Wnt signaling mainly includes the Wnt/β-catenin pathway, the noncanonical planar cell polarity (PCP) pathway, and the Wnt/calcium (Ca^2+^) pathway. In Wnt/β-catenin pathway, in the absence of Wnt signaling, β-catenin is phosphorylated in a destruction complex and ubiquitinated, leading to its degradation. Binding of Wnt to frizzled (Fz) and low density lipoprotein receptor related protein (LRP)-5/6 results in the activation of dishevelled (Dvl), which promotes β-catenin stabilization and nuclear translocation to act as a transcriptional coactivator of lymphoid enhancing factor-1 and T-cell factor-1 (LEF/TCF) transcription factors to activate the transcription of multiple target genes [4]. The Wnt/Ca^2+^-PCP pathway also appears to involve a Wnt ligand and Fz, but does not involve LRP-5/6 co-receptors and β-catenin. Activation of this pathway leads to the association of Dvl with Rac, Rho and Rho-associated kinase (ROCK), resulting in the restructuring of cytoskeleton.

Dapper, an antagonist of β-catenin, homolog 2 (DACT2) belongs to the DACT (Dpr/Frodo) gene family, located at 6q27, a region frequently associated with loss of heterozygosity in human cancers [5]. The DACT family consists of DACT1, 2 and 3 [6]. DACT family member and Dvl form a complex with Axin, GSK-3, CKI, and β-catenin, resulting in a decrease of activated β-catenin and suppression of target genes. These members also inhibit the activation of c-Jun N-terminal kinase (JNK) by Dvl. Thus, DACT genes are predicted to be potent cancer-associated genes [7].

In addition to genetic mutations in Wnt/β-catenin pathway components, epigenetic events also contribute to the abnormal activation of this signaling pathway in tumor cells. Epigenetic silencing of negative regulators of Wnt signaling is crucial for the aberrant activation of Wnt/β-catenin signaling in tumor pathogenesis [8–9]. For examples, promoter methylation of Secreted Frizzled-Related Proteins (SFRPs), Wnt Inhibitory Factor-1 (WIF-1), and DICKKOPFs (DKK-1, 2, 3) have been reported in multiple cancers [10–12].

*DACT1* expression has been shown to be significantly downregulated in hepatocellular carcinoma (HCC) [13], gastrointestinal stromal tumors [14], non-small cell lung cancer (NSCLC) [15] and breast cancer [16]. Dysregulated *DACT1* was associated with poor prognosis in non-small cell lung cancer patients [15]. DACT2 and DACT1 show 28.8% total-amino-acid identity. The expression level of DACT2 is reduced in some colorectal tumors [17]. However, little is known about the signaling function of DACT2 and its relevance to breast oncogenesis.

We previously identified DACT2 as a methylated target in our breast cancer methylome study. Here, we further examined DACT2 as a negative regulator of Wnt signaling and found that its transcription is repressed in breast cancer cell lines and primary tumors, which is associated with its promoter CpG methylation. The biological functions of DACT2 in breast cancer cells were assessed *in vitro* in the context of Wnt/β-catenin signaling.

## RESULTS

### Downregulation of *DACT2* in breast cell lines by promoter CpG methylation

**Figure 1:**
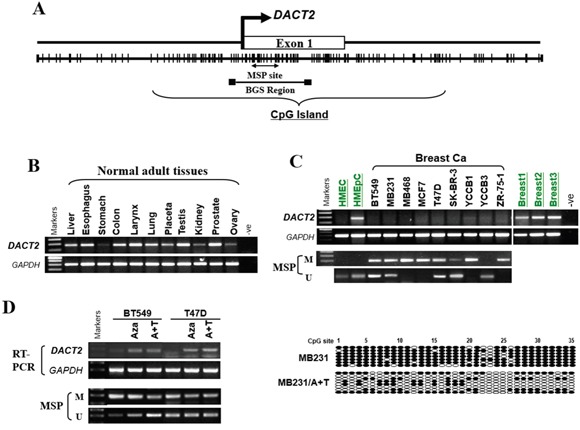
The expression and methylation status of *DACT2* in breast cancer cell lines and normal mammary tissues **A.** Schematic structure of the *DACT2* promoter CpG island (CGI). The white rectangle represents exon 1, and the CpG sites in the CGI are indicated with short black lines. **B.** Robust mRNA expression of *DACT2* in human normal adult tissues detected by semiquantitative RT-PCR, GAPDH as a control. **C.** Expression of *DACT2* in breast cancer cell lines, and the methylation status of *DACT2* in breast cancer and normal mammary epithelial cells. **D.** Pharmacological demethylation of the *DACT2* CGI by Aza (A) with or without TSA (T) induced its expression. *DACT2* expression before and after drug treatment was determined by RT–PCR, and demethylation was confirmed by MSP and BGS.

Promoter sequence analysis of the *DACT2* gene identified a typical CpG island spanning the proximal promoter and exon 1 regions (http://cpgislands.usc.edu/) (Figure 1A). We next performed RT-PCR analysis to examine *DACT2* expression in nine breast cancer cell lines. Semi-quantitative RT-PCR showed that *DACT2* was abundantly expressed in normal human tissues including breast, while silenced or downregulated in all breast cell lines analyzed (Figure 1B, 1C). Thus, the methylation status of *DACT2* promoter was examined. MSP showed that *DACT2* was methylated in 7 cell lines (BT549, MB231, MB468, MCF7, T47D, ZR-75-1 and YCCB1), with weak methylation in SK-BR-3 and no methylation in YCCB3 detected (Figure 1C). Pharmacological demethylation was used to assess whether promoter CpG methylation directly regulates *DACT2* expression. BT549 and T47D cells with methylated and silenced *DACT2* were treated with Aza with or without the histone deacetylase inhibitor TSA. Both treatments resulted in the upregulation of *DACT2* expression accompanied by a decrease in the methylated alleles of *DACT2* (Figure 1D). The results indicated that promoter methylation is a major mechanism of *DACT2* silencing in breast cancer cells. BGS of MB231 cells confirmed the results of MSP analysis, showing heavily methylated *DACT2* promoter alleles, while Aza treatment decreased its methylation in MB231 cells, leading to *DACT2* upregulation (Figure 1D).

### *DACT2* methylation in breast tumors and its correlation with clinical features

**Figure 2:**
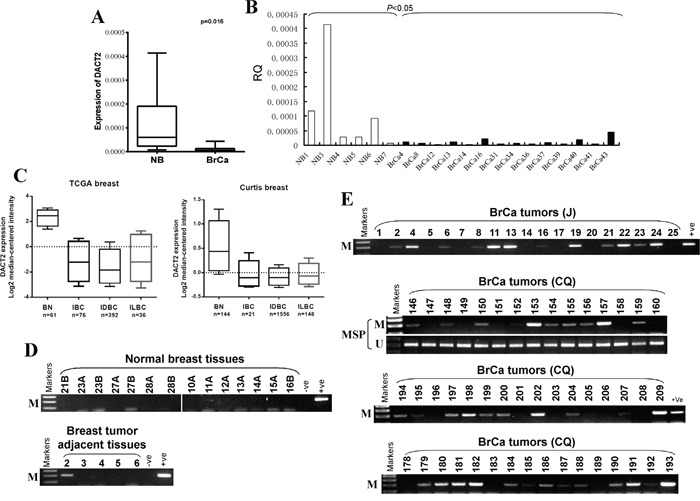
The expression and methylation status of *DACT2* in breast tumor tissues **A, B.** Expression of *DACT2* in human normal and breast tumor tissues detected by qRT-PCR, **C.** The expression of *DACT2* (median of expression intensity) in breast cancer from Oncomine database. **D.** Methylation of *DACT2* in normal breast tissues and breast tumor adjacent tissues. **E.** Representative images of methylation of the *DACT2* promoter in breast tumor tissues. M: methylated; U: unmethylated.

*DACT2* expression in human breast cancer samples was analyzed using the online database Oncomine and qRT-PCR. We found that the expression of *DACT2* mRNA in breast tumor specimens was significantly lower than that in non-tumor breast tissue specimens. The average level of *DACT2* mRNA expression in breast cancer tissues was 2.25-fold lower than that in adjacent non-cancerous tissues (*p*<0.05) (Figure 2A, 2B). TCGA breast studies demonstrated a significant 14.7-fold and 9.3-fold decrease in *DACT2* mRNA expression in Invasive Ductal Breast Carcinoma (IDBC) and Invasive Lobular Breast Carcinoma (ILBC), respectively, and an 11.2-fold decrease in Invasive Breast Carcinoma (IBC) compared to normal breast tissues (Figure 2C). Curtis Breast Statistics showed similar results (Figure 2C).

We further investigated *DACT2* methylation in primary tumors, surgical margin tissues and normal breast tissues. *DACT2* methylation was detected in 73% (107/147) of breast cancer tissues, 20% (1/5) of breast tumor adjacent tissues and none of normal breast tissues (Figure 2D, 2E; Table 1), suggesting the tumor-specific methylation of *DACT2* in breast cancer. We next analyzed the correlation between *DACT2* methylation and clinicopathological features of breast cancer patients, including age, tumor size, tumor grade, lymph node metastasis, and estrogen receptor (ER), progesterone receptor (PR) and HER2 status. However, no significant correlation between *DACT2* methylation and clinicopathological features was observed (Table 2).

**Table 1:** Methylation status of the *DACT2* promoter in primary breast tumors

Samples	*DACT2* promoter		Frequency of methylation
	methylated	unmethylated	
BrCa (n=147)	107	40	107/147 (73%)
BA (n=5)	1	4	1/5 (20%)
BNP (n= 14)	0	14	0/14 (0%)

Note: BrCa, breast cancer; BA, breast cancer adjacent tissues; BNP, breast normal tissues.

**Table 2:** Clinicopathological features of *DACT2* methylation in breast cancer

Clinicopathological features	Number(n=158)	*DACT2* promoter methylation status		*P* value
		methylated	unmethylated	
**Age**				0.602
≤40	13	11(85%)	2(15%)	
>40	103	74(72%)	29(28%)	
unknown	31	22(71%)	9(29%)	
**Grade**				0.431
I	7	5(71%)	2(29%)	
II	80	57(71%)	23(29%)	
III	7	7(100%)	0	
unknown	53	38(72%)	15(28%)	
**Tumor size**				0.289
<2.0 cm	43	36(84%)	7(16%)	
≥2.0 cm≤5.0cm	62	43(69%)	19(31%)	
>5.0cm	9	6(67%)	3(33%)	
unknown	33	22(67%)	11(33%)	
**Lymph node metastasis**				0.9
Positive	50	37(74%)	13(26%)	
Negative	64	47(73%)	17(27%)	
unknown	33	23(70%)	10(30%)	
**ER status**				0.479
Positive	52	36(69%)	16(31%)	
Negative	44	35(80%)	9(20%)	
unknown	51	36(71%)	15(29%)	
**PR status**				**0.048**
Positive	40	25(63%)	15(27%)	
Negative	56	47(84%)	9(16%)	
unknown	51	35(69%)	16(31%)	
**HER2 status**				0.606
>+++	9	6(67%)	3(33%)	
++	45	36(80%)	9(20%)	
<+	41	28(68%)	13(32%)	
unknown	52	37(71%)	15(29%)	
**p53 expression**				0.662
Positive	35	27(77%)	8(23%)	
Negative	44	30(68%)	14(32%)	
unknown	68	50(74%)	18(26%)	

### Re-expression of DACT2 inhibits cell growth and induces apoptosis in breast cancer cells

**Figure 3:**
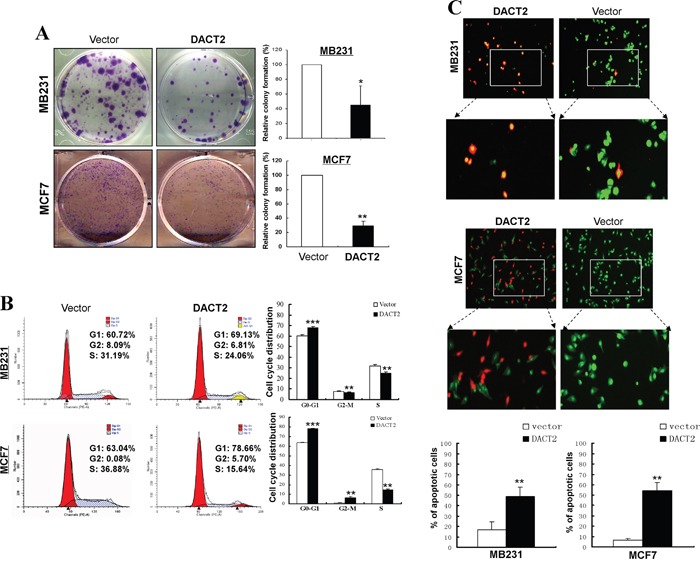
Growth inhibitory effect of DACT2 on breast cancer cell lines **A.** Representative colony formation assay in vector- and DACT2-expressing MB231 and MCF7 cells. The values are shown as the mean ± S.E. from three independent experiments (*p< 0.05, **p< 0.01). **B.** Effect of cell cycle distribution of vector- and DACT2-transfected MB231 and MCF7 cells was detected by flow cytometry analysis. Representative flow cytometry plots (left) and histograms of cell cycle alterations (right). **C.** Induction of apoptosis was detected by AO/EB assay.

To determine whether DACT2 suppresses cell growth in breast cancer, colony formation assays were performed using MB231 and MCF7 cells transfected with a DACT2-expressing plasmid or the empty vector. Cells transfected with DACT2 showed a dramatic decrease in colony numbers compared to control cells (Figure 3A). Moreover, MB231 cells ectopically expressing DACT2 inhibited cell proliferation by inducing a G1-phase cell cycle arrest and displayed condensed nuclei typical of apoptosis (Figure 3B, 3C). These results further demonstrated that DACT2 functions as a potential tumor suppressor in breast cancer.

### DACT2 reverses EMT through antagonizing β-catenin activity and inhibiting Akt/GSK-3β pathway in breast cells

**Figure 4:**
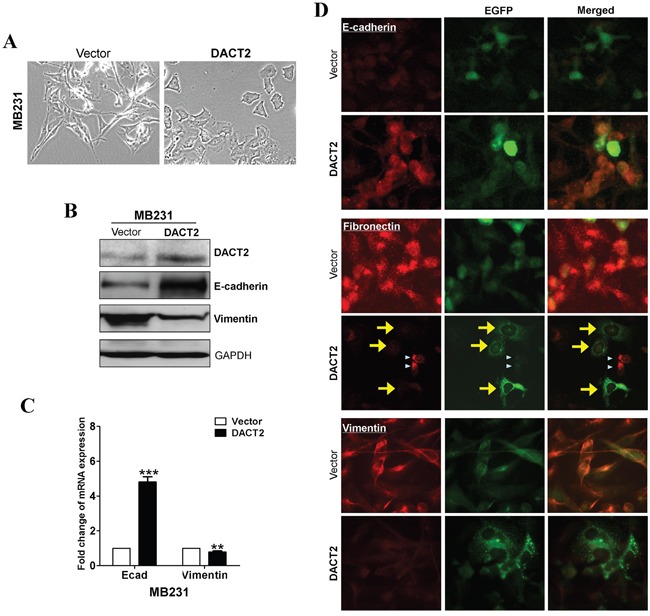
**A.** Morphology changes of MB231 cells transfected with DACT2 or empty vector by phase-contrast microscopy. **B.** Western blot analysis of E-cadherin and Vimentin. GAPDH was used as an internal control. **C.** qRT-PCR analysis of E-cadherin and Vimentin. β-actin was used as an internal control. **D.** Subcellular localization of EMT markers was detected by immunofluorescence staining.

**Figure 5:**
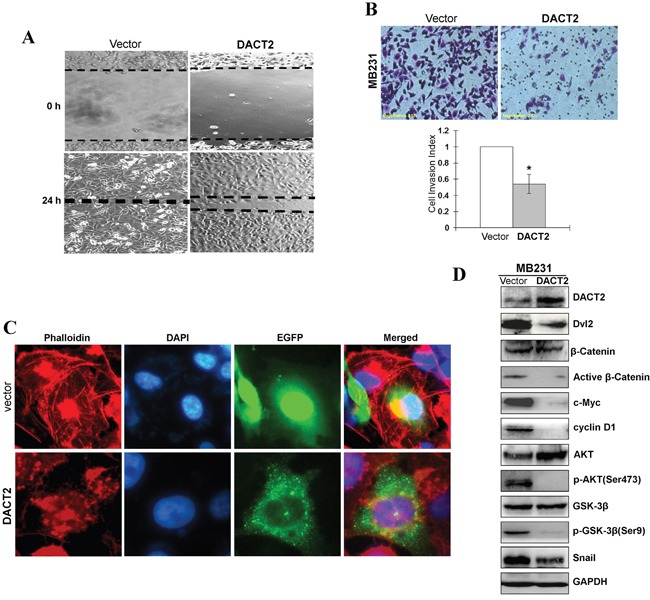
Ectopic expression of DACT2 inhibited the migration and invasion of breast tumor cells **A.** Representative images of wound healing assay. Photographs were taken at 0 and 24 h to determine the different mobility between Vector- and DACT2-transfected MB231 cells. **B.** Representative images of the transwell invasion assay. The pictures were taken at 24 h after seeding (magnification, ×200). The numbers of invaded cells were counted in seven representative high power fields per transwell. The values are shown as mean±SD. Three independent experiments were carried out in triplicate, *p<0.05. **C.** Cells transfected with DACT2 or empty vector were stained with FITC-phalloidin to observe the organization of actin fibers. Pictures were taken under 400× magnification. **D.** Ectopic expression of DACT2 in MB231 cells disrupted Wnt/β-catenin signaling and AKT/GSK-3β pathway. Western blot analysis of β-catenin and AKT, as well as their downstream targets in MB231 cells. GAPDH was used as an internal control.

We next examined whether epithelial-to-mesenchymal transition (EMT) in breast cancer cells is affected by DACT2 using the aggressive triple-negative breast cancer cell line MB231. The results showed that forced transfection of DACT2 effectively reversed EMT to mesenchymal-to- epithelial transition in MB231 cells, resulting in the upregulation of the epithelial marker E-cadherin and downregulation of the mesenchymal marker Vimentin (Figure 4A–4D). Activation of Wnt/β-catenin pathway and Akt/GSK-3 pathway play a central role in the maintenance of EMT. DACT family members in various species have previously been shown to interact with Dvl proteins through a highly conserved C-terminal motif and to negatively regulate β-catenin function. Therefore, we investigated whether DACT2 functions as a tumor suppressor by antagonizing Wnt/β-catenin signaling. Expression of DACT2 led to the downregulation of active β-catenin and Dvl2 in MB231 cells. Furthermore, overexpression of DACT2 in MB231 breast cancer cell lines resulted in a profound reduction of both total and phosphorylated forms of Akt and GSK-3β (Figure 5D).

### DACT2 suppresses breast cancer cell migration and invasion and induces actin cytoskeleton reorganization

EMT is a phenomenon that is accompanied by increased cell motility and invasion and involves loss of epithelial cell-cell junctions and actin cytoskeleton reorganization. The effect of DACT2 on the migration and invasiveness of breast cancer cells was analyzed using a wound-healing assay and Matrigel invasion chambers. The results of wound-healing assays showed that MB231 cells migrated into the scraped areas within 24 h, whereas DACT2 expression reduced wound closure by approximately 55% after 24 h (Figure 5A). Furthermore, DACT2-transfected cells had lower invasion rates than control cells (Figure 5B), suggesting that DACT2 attenuates wound-induced cell migration and inhibits the invasiveness of breast cancer cells. Cells transfected with DACT2 or empty vector were stained with FITC-phalloidin to observe the organization of actin fibers. Cells transfected with DACT2 displayed diffuse cytoplasmic actin, arranged irregularly, and much fewer thin stress fibers, whereas control group of cells present formation of actin stress fibers, supporting a role for DACT2 regulating actin cytoskeleton reorganization. (Figure 5C).

## DISCUSSION

Epigenetic disruption of tumor suppressor genes (TSGs) by promoter CpG methylation and histone modification is a key mechanism of gene deregulation in cancer [18–19]. DACT2 is frequently downregulated by promoter methylation in HCC [20] and colorectal tumors [17]. High DACT2 protein levels were correlated with better differentiation and better survival rates in HCC and esophageal squamous cell carcinoma patients [20–21]. Here, we showed that *DACT2* is expressed in normal breast tissues but frequently downregulated/silenced by promoter methylation in breast cancer cell lines and primary tumors, but not in normal breast tissues. No methylation was detected in the YCCB3 cell line with silenced *DACT2*, suggesting that histone modifications or other mechanisms are also involved in *DACT2* downregulation in this cell line. We showed the tumor-specific methylation of *DACT2* in breast cancer, which suggests its potential as a tumor marker. However, no obvious correlation between *DACT2* methylation and clinicopathological features was observed in breat cancer, although this needs to be further confirmed in a larger cohort study.

DACT family members have N-terminal leucine zipper domains and C-terminal PDZ binding motifs and can bind to many factors in both cytoplasmic and nuclear compartments [6, 22]. DACT2 interacts with PKC, Dvl3, β-catenin, PITX2 and Lef-1 [23]. Several studies have shown that DACT2 has distinct roles in different cells, indicating tissue type- or species- specific roles of DACT2. DACT2 in zebrafish regulates the noncanonical Wnt/Ca^2+^−PCP pathway [4], but also inhibits TGF-β/Nodal pathway [24] by targeting the TGF-β receptors ALK5 and ALK4, accelerating their lysosomal degradation [25], thus preventing the activation of Smad2/3. Mouse Dact2 antagonizes TGF-β signaling [26], as well as Wnt/β-catenin and Wnt/JNK signaling without directly altering β-catenin level [22, 27–28]. DACT2 remarkably suppressed TGF-β signaling via both proteasomal and lysosomal pathways in esophageal squamous cell carcinoma [21]. Recent studies further indicated that DACT2 could suppress Wnt signaling by inhibiting TCF/LEF in lung cancer [29]. However, in esophageal squamous cell carcinoma, DACT2 significantly decreases pSmad2/3 binding to their response elements, but has no effect on regulatory elements of canonical Wnt or other pathways [21].

Wnt/β-catenin and Akt/GSK-3 pathways have been well characterized to induce EMT in epithelial cell models [30]. Indeed, DACT2 re-expression significantly increased E-cadherin and decreased Vimentin levels in our study. EMT is associated with actin reorganization, tubular basement membrane disruption, and cell migration. DACT2 inhibited breast cancer cell proliferation by inducing apoptosis, and further suppressed tumor cell migration. We further showed that Dvl2 and activated β-catenin were inhibited by DACT2 re-expression in breast tumor cells. As expected, expression of p-AKT and p-GSK-3β dramatically decreased in MB231 cells upon DACT2 re-expression. Snail, which is tightly regulated by GSK-3β at the protein level, was also significantly downregulated.

In summary, DACT2 was found to be an antagonist to Wnt/β-catenin signaling and was frequently downregulated/silenced in breast cancer. Our results indicate that DACT2 acts as an important TSG for breast cancer, which may serve as a potential biomarker or therapeutic stargeting strategy for breast cancer.

## MATERIALS AND METHODS

### Cell lines, tumor samples and normal tissues

Breast cancer cell lines (BT549, MB231, MB468, MCF7, T47D, SK-BR-3, YCCB1, YCCB3 and ZR-75-1) were used. Normal breast tissue RNA samples were purchased commercially (Stratagene, La Jolla, CA; Millipore Chemicon, Billerica, MA; BioChain Institute, Hayward, CA). Primary tumor samples, paired surgical margin tissues and normal breast tissues were obtained from the First Affiliated Hospital of Chongqing Medical University [31–33]. All samples were reviewed and subjected to histological diagnosis by pathologists to ensure the percentage of tumor cells was ~70%. Clinical information was collected for most tumor samples. The study was approved by the ethics committee of the First Affiliated Hospital of Chongqing Medical University (Approval notice: 2010/2012(23)).

### Semiquantitative reverse transcription PCR (RT-PCR) and quantitative RT-PCR (qRT-PCR)

Total RNA was isolated using the TRIzol® Reagent (Invitrogen, Carlsbad, CA). Semiquantitative RT-PCR was performed in a final volume of 10 μl of reaction mixture containing 2 μl cDNA using Go-Taq (Promega, Madison, WI) [34]. *GAPDH* was amplified as the control. Primer sequences are listed in Table 1. Real-time PCR was performed using Thermo Fisher Maxima SYBR Green qPCR Master Mixes (Thermo-Fisher Scientific, former Fermentas, Schwerte, Germany). Thermal cycling reactions were performed using the 7500 Real-Time PCR System (Applied Biosystems, Foster City, CA). Relative expression levels of DACT2 in breast tissues were standardized to β-actin levels. Online cancer database Oncomine (http://www.oncomine.org) was used to examine the mRNA expression of DACT2 in normal versus breast tumor tissues. *p*-values for each group were calculated using Student's *t*-test. Standardized normalization techniques and statistical calculations are provided on the Oncomine website and published.

### 5-aza-2'-deoxycytidine (Aza) and trichostatin a treatment

Cell lines were treated with 10 mmol/l Aza (Sigma-Aldrich, St Louis, MO) for 3 days and further treated with 100 nmol/l trichostatin A (TSA, Cayman Chemical Co., Ann Arbor, MI) for 24 h.

### Methylation-specific PCR (MSP) and bisulfite genomic sequencing (BGS)

Genomic DNA was extracted from cell pellets, normal tissues and tumors using the DNAzol® Reagent (Invitrogen, Carlsbad, CA). Bisulfite modification of DNA and MSP were performed as described previously [35–36]. Bisulfite-treated DNA was amplified by MSP with *DACT2* methylation-specific primers (Table 3) using AmpliTaq-Gold DNA Polymerase (Applied Biosystems, Foster City, CA). Methylated and unmethylated MSP primer sets target the same CpG sites in the DACT2 promoter, and do not amplify genomic DNA with no bisulfite treatment. For BGS, bisulfite-treated DNA was amplified using a BGS primer (Table 3). The PCR products were then cloned into a pCR4-Top vector (Invitrogen, Carlsbad, CA). Eight to twelve colonies were randomly chosen and sequenced by Beijing Genomics Institute.

**Table 3:** List of primers used in this study

PCR	Primer	Sequence (5'-3')	Product size (bp)	PCR Cycles	Annealing temperature (°C)
**MSP**	DACT2 M1	CGTGTAGATTTCGTTTTTCGC	**200**	**40**	**60**
	DACT2 M2	CCGAAAATCCGCCCGACG			
	DACT2 U1	TGTGTGTAGATTTTGTTTTTTGT	**203**	**40**	**58**
	DACT2 U2	CCCCAAAAATCCACCCAACA			
**RT-PCR**	DACT2F	AGCCGTGGGGCACATTCTG	**173**	**35**	**55**
	DACT2R	CCAGGTCCTGCCGATACTTG			
**BGS**	DACT2-BGS1	GGTTATAGATTTTAGTTTATTTTGG	**249**	**40**	**60**
	DACT2-BGS2	CTACAACTCCTACAACCCC			

### Immunofluorescence staining

Cells transfected with pcDNA3.1-DACT2 or pcDNA3.1 plasmid were seeded in six-well tissue culture plates containing glass coverslips. The cells were washed with PBS, fixed with precooled methanol at −20°C for 10 min, permeabilized with 0.5% Triton X-100 for 5 min and blocked with 5% normal goat serum at room temperature for 30-40 min. Cells were incubated with primary antibodies diluted in TBST at 4°C overnight, washed twice with PBS, and then incubated with Alexa Fluor 594- or 488-conjugated goat anti-rabbit or anti-mouse secondary antibody (Invitrogen Molecular Probes, Carlsbad, Ca) for an additional 30 min. Nuclei were counterstained with 4,6-diamidino- 2- phenylindole (DAPI) (Roche, Palo Alto, CA). To analyze the effects of DACT2 on F-actin, the cells were fixed with 4% paraformaldehyde for 20 min at room temperature and stained by rhodamine-labeled phalloidin (Invitrogen Molecular Probes). The slides were visualized with an Olympus BX51 microscope (Olympus Corporation, Tokyo, Japan) under 400× magnification and the images were captured with a camera.

### Colony formation assay

Colony formation assays were performed as described previously [16]. MB231 and MCF7 cells were plated in six-well plates and transfected with 4 μg pcDNA3.1-DACT2 or pcDNA3.1 plasmid using Lipofectamine 2000 (Invitrogen, Carlsbad, CA). At 48 h after transfection, cells were collected, re-plated in six-well plates and selected for 14 days in the presence of G418 (1.2 mg/ml for MB231, 0.8 mg/ml for MCF7). Surviving colonies (≥50 cells/colony) were stained with Gentian Violet and counted. All experiments were performed three times.

### Wound healing assay and cell invasion assay

Stably transfected cells were selected using G418, and then cultured in six-well plates until confluent. After scratching the monolayer, cells were photographed at 0, 12 and 24 h under a 10× objective (Olympus Corporation, Tokyo, Japan). Cell invasion assay was performed using 24-well culture plates (Millipore, Billerica, MA) with inserts of 8-μm pore membranes pre-coated with Matrigel (BD Bioscience, San Jose, CA). Briefly, 1×10^4^ cells were seeded in each well. The lower compartment was filled with cell culture medium supplemented with 15% fetal bovine serum. After 24 h, invaded cells on the bottom surface were fixed with methanol and stained with 0.1% Crystal Violet. The invaded cells were counted and photographed under a microscope. Seven fields per membrane were counted in each group. Each experiment was performed twice.

### Flow cytometry analysis of cell cycle status and apoptosis assay

To assess cell cycle status, MB231 cells and MCF7 cells were seeded in six-well plates and transfected with 4 μg of DACT2 or empty vector using Lipofectamine 2000 (Invitrogen, Carlsbad, CA) following the manufacturer's protocol. After 48 h, cells were harvested using 0.1% trypsin and washed with PBS, then fixed in ice-cold 70% ethanol for 1 h, and treated with 100 μl of 50 mg/l propidium iodide for 30 min at 4°C in the dark. Data were analyzed with the CELL Quest software (BD Biosciences, San Jose, CA). Acridine orange/ethidium bromide (AO/EB) staining was used for apoptosis analysis. Briefly, the transfected cells were replated in six-well plates containing glass coverslips. After 24 h, cells were washed with phosphate-buffered saline (PBS) then stained with AO/EB for 5 min and visualized immediately under a fluorescence microscope (LEICA CTR4000B). The percentage of apoptotic cells was then calculated.

### Western blotting

Transfected cells were washed with ice cold PBS and lysed with lysis buffer (Pierce, Thermo Scientific, Cramlington, UK) containing a protease inhibitor cocktail (Sigma Aldrich, St. Louis, MO). The lysate was centrifuged at 4°C for 10 min at 10,000 *g* and the supernatant was collected. A total of 50 μg of protein lysate for each sample was separated using sodium dodecyl sulfate/polyacrylamide gel electrophoresis. The lysates were then transferred to polyvinylidene fluoride membranes for antibody incubation. After blocking with 5% nonfat milk and 0.1% Tween 20 in TBS, the membranes were incubated with the following primary antibody overnight at 4°C: β-catenin (1:1000, Cell Signaling, #2677), active β-catenin (1:1000, Cell Signaling, #4270), cyclinD1 (1:1000, Epitomics, #2261), c-Myc (1:10000, Epitomics, #1472-1), p-AKT (Ser473) (1:1000, Cell Signaling #4060), p-GSK-3β (Ser9) (1:1000, Cell Signaling #9323) and GAPDH (1:2000, Epitomics, #2261) was used as control. Next day the membranes were washed and incubated with secondary antibodies. Bands were visualized using the enhanced chemiluminescence detection system.

### Statistical analysis

Statistical analyses were performed using the χ^2^ test and Fisher's exact test to determine the *p* value. *p*<0.05 was considered statistically significant.
